# Supplementary material for: Prohibitin Links Cell Cycle, Motility and Invasion in Prostate Cancer Cells
Source: Int J Mol Sci. 2023 Jun 8;24(12):9919. doi: 10.3390/ijms24129919 (PMC10298516; doi:10.3390/ijms24129919)
Supplement: Supplementary file 1 [file ijms-24-09919-s001.zip › ijms-2201646-supplementary.pdf]

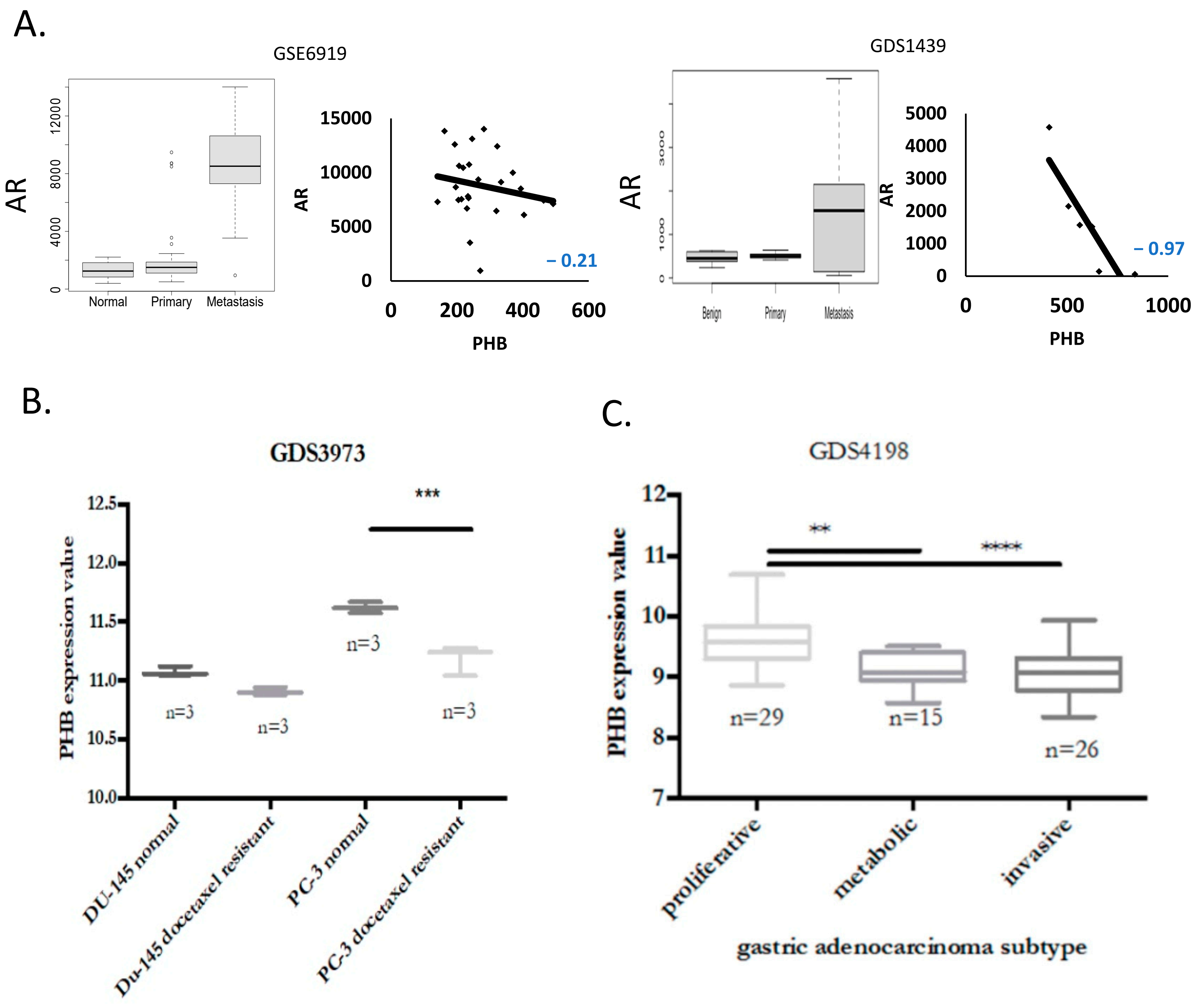

Supplemental Figure S1.

A, Boxplots showing AR expression values from the GSE6919 and GDS1439 datasets. Data is separated into normal prostate tissue (n=17), primary prostate cancer (n=66) and metastatic prostate cancer (n=25). Graph on left hand side shows the regression analysis of AR versus PHB expression. **B**, Boxplots showing the PHB expression values from GDS3973 (docetaxel-resistant prostate cancer cell lines. **C**, Boxplots showing PHB expression from the GDS4198 dataset (gastric adenocarcinoma). ** $p \leq 0.01$, *** $p \leq 0.001$, **** $p \leq 0.0001$.

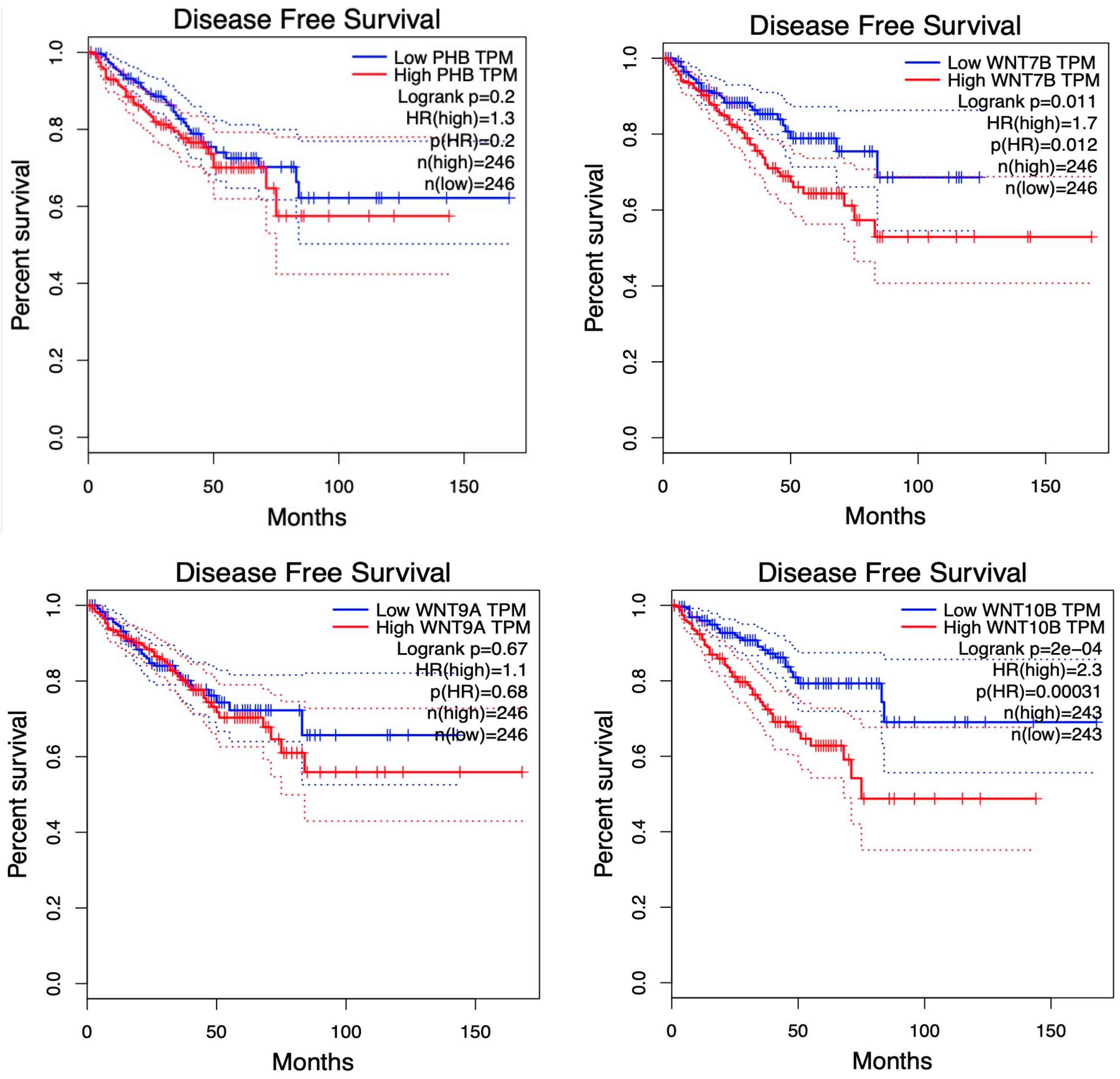

Supplemental Figure S2.

Kaplan-meier disease free survival plots for PHB, WNT7B, 9A and 10B. Dotted lines represent the 95% confidence interval. Red is high expression, blue is low expression. Data from Gepia Tang, Z. et al. (2017) GEPIA: a web server for cancer and normal gene expression profiling and interactive analyses. Nucleic Acids Res, 10.1093/nar/gkx247.

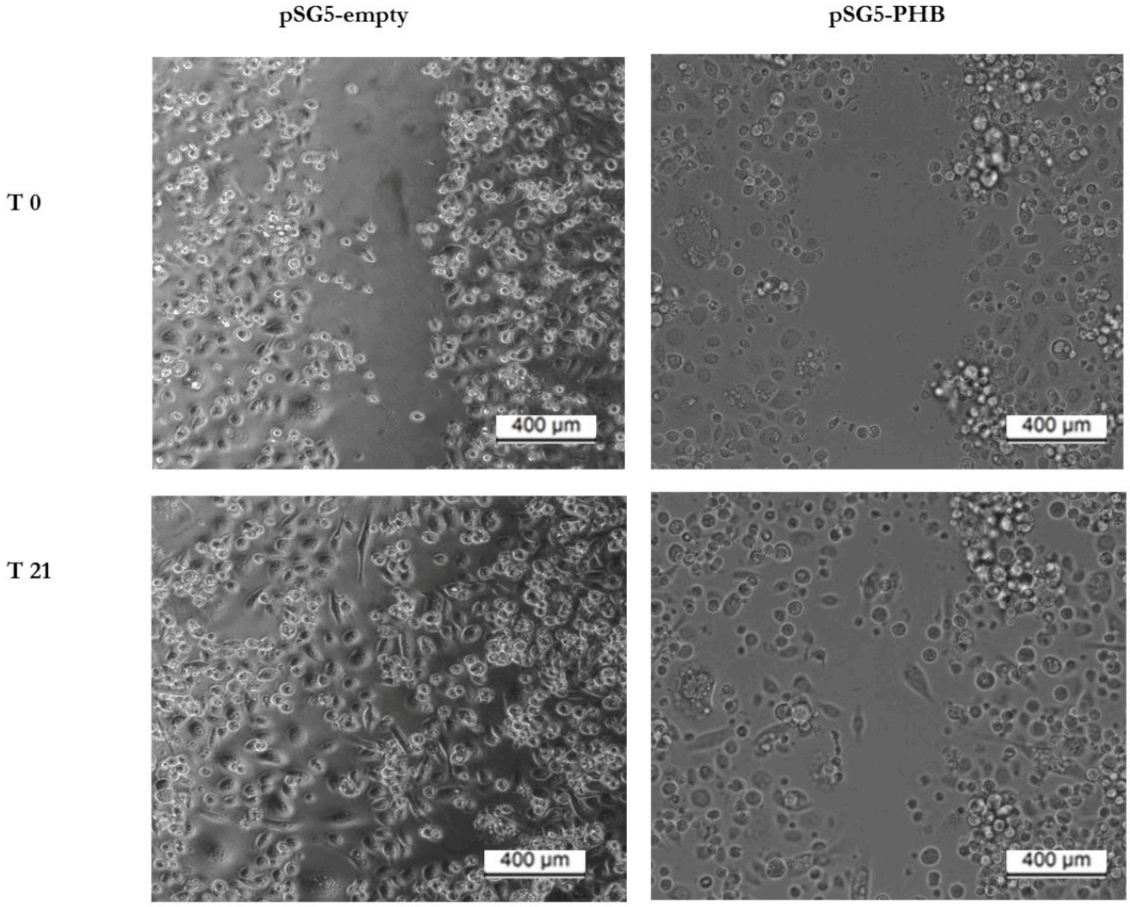

Supplemental Figure S3.

Wound healing assay using PC3 cells, either transfected with empty or pSG5-PHB expression vector over 21 hours.

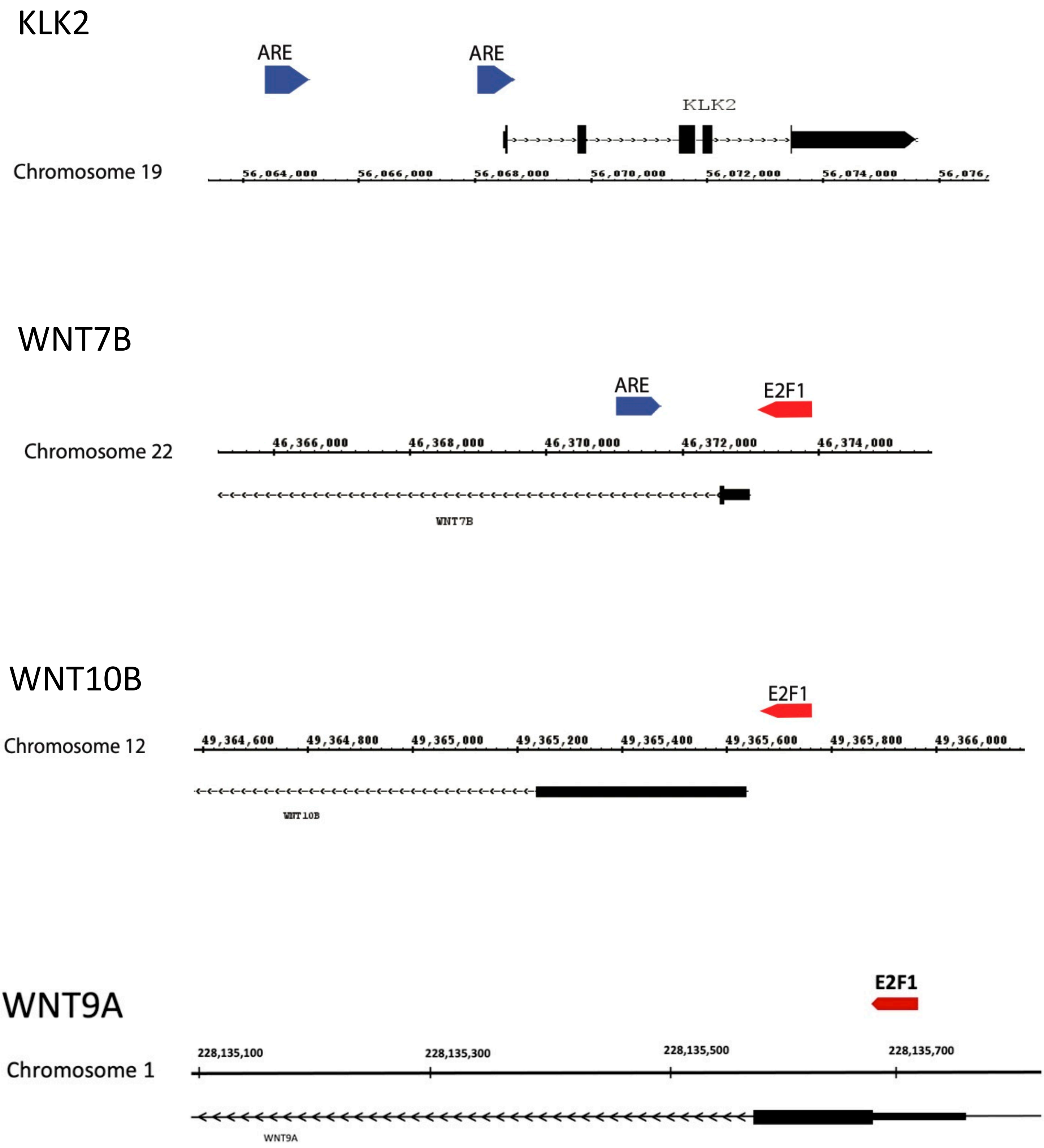

Supplemental Figure S4.

Analysis of E2F1 & AR binding sites in the WNT7B, 9A and 10B genes, including promoter regions. The androgen-regulated KLK2 gene is given for comparison. Data taken from genome browser (https://genome.ucsc.edu/), GSE28219 and Alggen (https://Alggen.lsi.upc).

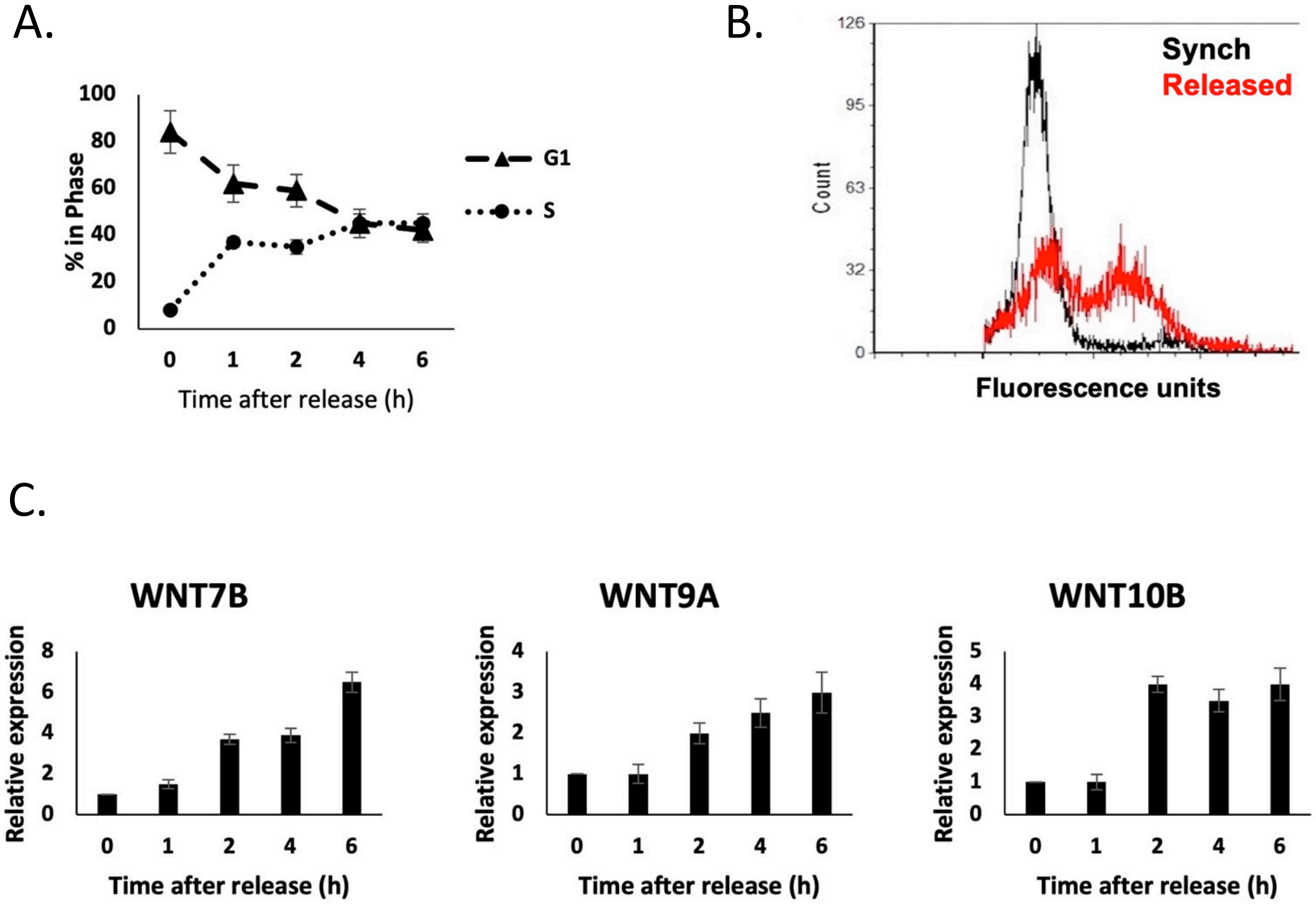

Supplemental Figure S5.

A, G1 and S phase % of HeLa cells either synchronised in a thymidine/aphidicolin block and then released into normal media for 0-6 hours. **B**, Cell cycle distribution of HeLa cells either synchronised with thymidine/aphidicolin and then released into normal media for 6 hours. **C**, Q-PCR analysis of WNT7B, 9A and 10B from HeLa cells released from thymidine/aphidicolin block. Normalised to *GAPDH*, *RPL19* and *β-actin* expression.
